# Supplementary material for: One-step multiplex PCR assay for identification of Mycobacterium kansasii complex species
Source: Microbiol Spectr. 2026 Mar 5;14(4):e03267-25. doi: 10.1128/spectrum.03267-25 (PMC13055360; doi:10.1128/spectrum.03267-25)
Supplement: Supplemental material — Tables S1 to S3; Fig. S1 to S3. [file spectrum.03267-25-s0001.pdf]

SUPPLEMENTAL TABLES

Suppl. Tab. 1. Isolates used in the *in vitro* experiments of this study.

No.	Species	Source	Strain ID	Collection	mPCR-based species
			***Mycobacterium kansasii* complex reference strains**		
1	*M. kansasii*	reference strain	ATCC12478	ATCC[1]	*M. kansasii*
2	*M. persicum*	reference strain	B11063838	ZMM IM UW[2]	*M. persicum*
3	*M. pseudokansasii*	reference strain	MK142	DSMZ[3]	*M. pseudokansasii*
4	*M. ostraviense*	reference strain	241/15	ZMM IM UW	*M. ostraviense*
5	*M. innocens*	reference strain	MK13	DSMZ	*M. innocens*
6	*M. attenuatum*	reference strain	MK41	DSMZ	*M. attenuatum*
7	*M. gastri*	reference strain	DSM43505	DSMZ	*M. gastri*
			***Mycobacterium kansasii* complex**		
1	*M. kansasii*	clinical	ATCC25221	ZMM IM UW	*M. kansasii*
2	*M. kansasii*	clinical	2082/12	ZMM IM UW	*M. kansasii*
3	*M. kansasii*	clinical	429/13	ZMM IM UW	*M. kansasii*
4	*M. kansasii*	clinical	AUS13	ZMM IM UW	*M. kansasii*
5	*M. kansasii*	clinical	N1	ZMM IM UW	*M. kansasii*
6	*M. kansasii*	clinical	N3	ZMM IM UW	*M. kansasii*
7	*M. kansasii*	clinical	N4	ZMM IM UW	*M. kansasii*
8	*M. kansasii*	clinical	N6	ZMM IM UW	*M. kansasii*
9	*M. kansasii*	clinical	N8	ZMM IM UW	*M. kansasii*
10	*M. kansasii*	clinical	N9	ZMM IM UW	*M. kansasii*
11	*M. kansasii*	clinical	N10	ZMM IM UW	*M. kansasii*
12	*M. kansasii*	clinical	N11	ZMM IM UW	*M. kansasii*
13	*M. kansasii*	clinical	N13	ZMM IM UW	*M. kansasii*
14	*M. kansasii*	clinical	N15	ZMM IM UW	*M. kansasii*
15	*M. kansasii*	clinical	N16	ZMM IM UW	*M. kansasii*
16	*M. kansasii*	clinical	N20	ZMM IM UW	*M. kansasii*
17	*M. kansasii*	clinical	N24	ZMM IM UW	*M. kansasii*
18	*M. kansasii*	clinical	N26	ZMM IM UW	*M. kansasii*
19	*M. kansasii*	clinical	N28	ZMM IM UW	*M. kansasii*
20	*M. kansasii*	clinical	N29	ZMM IM UW	*M. kansasii*
21	*M. kansasii*	clinical	N30	ZMM IM UW	*M. kansasii*
22	*M. kansasii*	clinical	N31	ZMM IM UW	*M. kansasii*
23	*M. kansasii*	clinical	N35	ZMM IM UW	*M. kansasii*
24	*M. kansasii*	clinical	N36	ZMM IM UW	*M. kansasii*
25	*M. kansasii*	clinical	N38	ZMM IM UW	*M. kansasii*
26	*M. kansasii*	clinical	NLA001000449	ZMM IM UW	*M. kansasii*
27	*M. kansasii*	clinical	NLA001000927	ZMM IM UW	*M. kansasii*
28	*M. kansasii*	clinical	NJH6	ZMM IM UW	*M. kansasii*
29	*M. kansasii*	clinical	NJH7	ZMM IM UW	*M. kansasii*
30	*M. kansasii*	clinical	NJH8	ZMM IM UW	*M. kansasii*
31	*M. kansasii*	clinical	NJH9	ZMM IM UW	*M. kansasii*
32	*M. kansasii*	clinical	NJH10	ZMM IM UW	*M. kansasii*
33	*M. kansasii*	clinical	NJH11	ZMM IM UW	*M. kansasii*
34	*M. kansasii*	clinical	NJH12	ZMM IM UW	*M. kansasii*
35	*M. kansasii*	clinical	NJH13	ZMM IM UW	*M. kansasii*
36	*M. kansasii*	clinical	NJH14	ZMM IM UW	*M. kansasii*
37	*M. kansasii*	clinical	NJH15	ZMM IM UW	*M. kansasii*
38	*M. kansasii*	clinical	28	ZMM IM UW	*M. kansasii*
39	*M. kansasii*	clinical	40	ZMM IM UW	*M. kansasii*

No.	Species	Source	Strain ID	Collection	mPCR-based species
		***Mycobacterium kansasii* complex**			
40	*M. kansasii*	clinical	42	ZMM IM UW	*M. kansasii*
41	*M. kansasii*	clinical	69	ZMM IM UW	*M. kansasii*
42	*M. kansasii*	clinical	70	ZMM IM UW	*M. kansasii*
43	*M. kansasii*	clinical	83	ZMM IM UW	*M. kansasii*
44	*M. kansasii*	clinical	132	ZMM IM UW	*M. kansasii*
45	*M. kansasii*	clinical	159	ZMM IM UW	*M. kansasii*
46	*M. kansasii*	clinical	195	ZMM IM UW	*M. kansasii*
47	*M. kansasii*	clinical	224	ZMM IM UW	*M. kansasii*
48	*M. kansasii*	clinical	257	ZMM IM UW	*M. kansasii*
49	*M. kansasii*	clinical	322	ZMM IM UW	*M. kansasii*
50	*M. kansasii*	clinical	419	ZMM IM UW	*M. kansasii*
51	*M. kansasii* IB	clinical	K4[#]	ZMM IM UW	*M. kansasii*
52	*M. kansasii* I/II	clinical	NLA00100521[#]	ZMM IM UW	*M. kansasii*
53	*M. kansasii* IIB	clinical	K14[#]	ZMM IM UW	*M. persicum*
54	*M. kansasii* IIB	clinical	K19[#]	ZMM IM UW	*M. persicum*
55	*M. persicum*	clinical	AUS4	ZMM IM UW	*M. persicum*
56	*M. persicum*	clinical	AUS18	ZMM IM UW	*M. persicum*
57	*M. persicum*	clinical	B11073207	ZMM IM UW	*M. persicum*
58	*M. persicum*	clinical	H-47	ZMM IM UW	*M. persicum*
59	*M. persicum*	clinical	N14	ZMM IM UW	*M. persicum*
60	*M. persicum*	clinical	N18	ZMM IM UW	*M. persicum*
61	*M. persicum*	clinical	N27	ZMM IM UW	*M. persicum*
62	*M. persicum*	clinical	N34	ZMM IM UW	*M. persicum*
63	*M. persicum*	clinical	N5	ZMM IM UW	*M. persicum*
64	*M. persicum*	clinical	N63	ZMM IM UW	*M. persicum*
65	*M. persicum*	clinical	N78	ZMM IM UW	*M. persicum*
66	*M. persicum*	clinical	NLA0010011128	ZMM IM UW	*M. persicum*
67	*M. persicum*	clinical	S14	ZMM IM UW	*M. persicum*
68	*M. pseudokansasii*	clinical	174/15	ZMM IM UW	*M. pseudokansasii*
69	*M. pseudokansasii*	clinical	4/15 (=14/15)	ZMM IM UW	*M. pseudokansasii*
70	*M. pseudokansasii*	clinical	AUS5	ZMM IM UW	*M. pseudokansasii*
71	*M. pseudokansasii*	clinical	AUS20	ZMM IM UW	*M. pseudokansasii*
72	*M. pseudokansasii*	clinical	N17	ZMM IM UW	*M. pseudokansasii*
73	*M. pseudokansasii*	clinical	N22	ZMM IM UW	*M. pseudokansasii*
74	*M. pseudokansasii*	clinical	N23	ZMM IM UW	*M. pseudokansasii*
75	*M. pseudokansasii*	clinical	N25	ZMM IM UW	*M. pseudokansasii*
76	*M. pseudokansasii*	clinical	N32	ZMM IM UW	*M. pseudokansasii*
77	*M. pseudokansasii*	clinical	N33	ZMM IM UW	*M. pseudokansasii*
78	*M. pseudokansasii*	clinical	N57	ZMM IM UW	*M. pseudokansasii*
79	*M. pseudokansasii*	clinical	N64	ZMM IM UW	*M. pseudokansasii*
80	*M. pseudokansasii*	clinical	N67	ZMM IM UW	*M. pseudokansasii*
81	*M. pseudokansasii*	clinical	UK6	ZMM IM UW	*M. pseudokansasii*
82	*M. ostraviense*	clinical	N68	ZMM IM UW	*M. ostraviense*
83	*M. ostraviense*	clinical	N73	ZMM IM UW	*M. ostraviense*
84	*M. ostraviense*	environmental	1010001458	ZMM IM UW	*M. ostraviense*
85	*M. innocens*	clinical	49/11	ZMM IM UW	*M. innocens*
86	*M. innocens*	clinical	N2	ZMM IM UW	*M. innocens*
87	*M. innocens*	environmental	1010001454	ZMM IM UW	*M. innocens*
88	*M. innocens*	environmental	1010001493	ZMM IM UW	*M. innocens*
89	*M. attenuatum*	clinical	AUS8	ZMM IM UW	*M. attenuatum*
90	*M. attenuatum*	clinical	AUS10	ZMM IM UW	*M. attenuatum*
91	*M. attenuatum*	clinical	AUS15	ZMM IM UW	*M. attenuatum*

No.	Species	Source	Strain ID	Collection	mPCR-based species
		Mycobacterium kansasii **complex**			
92	*M. attenuatum*	clinical	AUS16	ZMM IM UW	*M. attenuatum*
93	*M. attenuatum*	clinical	AUS24	ZMM IM UW	*M. attenuatum*
94	*M. attenuatum*	clinical	AUS28	ZMM IM UW	*M. attenuatum*
95	*M. attenuatum*	clinical	F2	ZMM IM UW	*M. attenuatum*
96	*M. attenuatum*	clinical	N19	ZMM IM UW	*M. attenuatum*
97	*M. attenuatum*	clinical	N61	ZMM IM UW	*M. attenuatum*
98	*M. attenuatum*	clinical	NLA001001166	ZMM IM UW	*M. attenuatum*
		**NTM other-than-MKC**			
1	*M. abscessus*	clinical	MAB1	ZMM IM UW	non-MKC
2	*M. abscessus*	clinical	MAB2	ZMM IM UW	non-MKC
3	*M. abscessus* subsp. *bolletii*	clinical	MAB3	ZMM IM UW	non-MKC
4	*M. abscessus* subsp. *massiliense*	clinical	MAM2B	ZMM IM UW	non-MKC
5	*M. avium*	clinical	MAV4	ZMM IM UW	non-MKC
6	*M. chelonae* subsp. *chelonae*	clinical	MCC5	ZMM IM UW	non-MKC
7	*M. chelonae*	environmental	J56	ZMM IM UW	non-MKC
8	*M. conspicuum*	reference strain	DSM44136	DSMZ	non-MKC
9	*M. fortuitum* subsp. *fortuitum*	reference strain	MF4	ZMM IM UW	non-MKC
10	*M. fortuitum*	environmental	IDUB17	ZMM IM UW	non-MKC
11	*M. gordonae*	clinical	N37	ZMM IM UW	non-MKC
12	*M. gordonae*	clinical	N43	ZMM IM UW	non-MKC
13	*M. gordonae*	environmental	IDUB5	ZMM IM UW	non-MKC
14	*M. gordonae*	environmental	IDUB22	ZMM IM UW	non-MKC
15	*M. gordonae*	environmental	IDUB26	ZMM IM UW	non-MKC
16	*M. gordonae*	environmental	IDUB27	ZMM IM UW	non-MKC
17	*M. gordonae*	environmental	IDUB28	ZMM IM UW	non-MKC
18	*M. intracellulare*	clinical	AUS19	ZMM IM UW	non-MKC
19	*M. immunogenum*	environmental	J63	ZMM IM UW	non-MKC
20	*M. riyadhense*	reference strain	DSM45176	DSMZ	non-MKC
21	*M. malmoense*	environmental	IDUB31	ZMM IM UW	non-MKC
22	*M. massiliense*	clinical	MK33	ZMM IM UW	non-MKC
23	*M. massiliense*	clinical	MK34	ZMM IM UW	non-MKC
24	*M. marinum*	reference strain	DSM44344	DSMZ	non-MKC
25	*M. paraterrae*	environmental	IDUB9	ZMM IM UW	non-MKC
26	*M. peregrinum*	environmental	IDUB25	ZMM IM UW	non-MKC
27	*M. phlei*	reference strain	JCM5865	JCM[4]	non-MKC
28	*M. porcinum*	clinical	MP6	ZMM IM UW	non-MKC
29	*M. salmoniphilum*	environmental	J113	ZMM IM UW	non-MKC
30	*M. septicum*	environmental	IDUB24	ZMM IM UW	non-MKC
31	*M. septicum*	environmental	J101	ZMM IM UW	non-MKC
32	*M. septicum*	environmental	J125	ZMM IM UW	non-MKC
33	*M. smegmatis*	reference strain	JCM5866	JCM	non-MKC
34	*M. szulgai*	reference strain	DSM44166	DSMZ	non-MKC
35	*M. terrae*	reference strain	JCM12143	JCM	non-MKC
		M. tuberculosis **complex**			
1	*M. bovis* BCG	reference strain	BCG	Pasteur Insitute	non-MKC
2	*M. tuberculosis* H37Rv	reference strain	ATCC27294	ATCC	non-MKC
3	*M. tuberculosis*	clinical	DLT97	ZMM IM UW	non-MKC

[1] ATCC – American Type Culture Collection; [2] ZMM IM UW – Department of Medical Microbiology, Institute of Microbiology, University of Warsaw (Zakład Mikrobiologii Medycznej, Instytut Mikrobiologii, Uniwersytet Warszawski); [3] DSMZ – German Collection of Microorganisms and Cell Cultures GmbH (Deutsche Sammlung von Mikroorganismen und Zellkulturen GmbH); [4] JCM – Japan Collection of Microorganisms. [#] Atypical strains.

Suppl. Tab. 2. Sequences used of the bioinformatic analyses.

No.	GenBank acc. no.	Species name	Strain ID*
1	GCF_000157895.3	*M. kansasii*	ATCC 12478 [T]
2	GCF_001632965.1	*M. kansasii*	1010001495
3	GCF_900565985.1	*M. kansasii*	MK22
4	GCF_900566155.1	*M. kansasii*	MK40
5	GCF_900565995.1	*M. kansasii*	MK7
6	GCF_002086895.1	*M. kansasii*	5MK
7	GCF_002085775.1	*M. kansasii*	6MK
8	GCF_002085625.1	*M. kansasii*	1MK
9	GCF_002085645.1	*M. kansasii*	4MK
10	GCF_002085795.1	*M. kansasii*	10MK
11	GCF_002085835.1	*M. kansasii*	9MK
12	GCF_002085815.1	*M. kansasii*	11MK
13	GCF_002705785.1	*M. kansasii*	K4
14	GCF_002705865.1	*M. kansasii*	K14
15	GCF_002705825.1	*M. kansasii*	K19
16	GCF_002920655.1	*M. kansasii*	BR3657
17	GCF_002920735.1	*M. kansasii*	BR6849
18	GCF_000715375.1	*M. kansasii*	SMC1
19	GCF_002920805.1	*M. kansasii*	BR8837
20	GCF_002920665.1	*M. kansasii*	BR6498
21	GCF_002003625.1	*M. kansasii*	Nov-13
22	GCF_002920705.1	*M. kansasii*	BR6884
23	GCF_002920795.1	*M. kansasii*	BR10742
24	GCF_002920775.1	*M. kansasii*	BR1580
25	GCF_002920815.1	*M. kansasii*	BR4404
26	GCF_002920875.1	*M. kansasii*	BR8839
27	GCF_002920675.1	*M. kansasii*	BR7287
28	GCF_002920755.1	*M. kansasii*	BR10953
29	GCF_002920845.1	*M. kansasii*	BR8835
30	GCF_002003585.1	*M. kansasii*	Nov-69
31	Kaust I	*M. kansasii*	-
32	GCF_015471985.1	*M. kansasii*	MKAN13-GN
33	GCF_000523615.1	*M. kansasii*	662
34	GCF_002086675.1	*M. persicum*	AFPC-000227 [T]
35	GCF_900566005.1	*M. persicum*	MK15
36	GCF_900566035.1	*M. persicum*	MK4
37	GCA_002086915.1	*M. persicum*	12MK
38	GCA_002086925.1	*M. persicum*	3MK
39	GCA_002086935.1	*M. persicum*	7MK
40	GCA_002086855.1	*M. persicum*	8MK
41	GCF_001632975.1	*M. persicum*	1010001469
42	GCF_002705895.1	*M. persicum*	H47
43	GCF_002705835.1	*M. persicum*	H48
44	GCF_900566015.1	*M. persicum*	MK42
45	Kaust II	*M. persicum*	-
46	GCF_902825395.1	*M. persicum*	CSURQ1465
47	GCF_001632915.1	*M. pseudokansasii*	1010001468

No.	GenBank acc. no.	Species name	Strain ID*
48	GCF_900566075.1	*M. pseudokansasii*	MK142 [T]
49	GCF_900566045.1	*M. pseudokansasii*	MK21
50	GCF_900566025.1	*M. pseudokansasii*	MK35
51	GCA_002705885.1	*M. pseudokansasii*	14_15
52	GCA_002705935.1	*M. pseudokansasii*	174_15_11
53	GCA_000524055.1	*M. pseudokansasii*	732
54	Kaust III	*M. pseudokansasii*	-
55	GCF_001632895.1	*M. ostraviense*	1010001458
56	GCF_002705925.1	*M. ostraviense*	241/15 [T]
57	Kaust IV	*M. ostraviense*	-
58	GCF_001632885.1	*M. innocens*	1010001493
59	GCF_001632905.1	*M. innocens*	1010001454
60	GCF_900566055.1	*M. innocens*	MK13 [T]
61	GCA_002705965.1	*M. innocens*	49_11
62	Kaust V	*M. innocens*	-
63	GCF_900566065.1	*M. attenuatum*	MK136
64	GCF_900566105.1	*M. attenuatum*	MK191
65	GCF_900566085.1	*M. attenuatum*	MK41 [T]
66	GCA_002086865.1	*M. attenuatum*	NLA001001166
67	GCF_002102175.1	*M. gastrii*	DSM 43505 [T]
68	GCF_000253355.1	*M. africanum*	GM041182
69	GCF_002982335.1	*M. africanum*	ATCC 25420
70	GCF_000729745.1	*M. bovis*	09-1191
71	GCF_005156105.1	*M. bovis*	Danish 1331
72	GCF_000338715.2	*M. bovis*	Korea 1168P
73	GCF_000010685.1	*M. bovis*	Tokyo 172
74	GCF_001274555.1	*M. bovis*	Russia 368
75	GCF_002975475.1	*M. bovis*	2002/0476
76	GCF_001078615.1	*M. bovis*	1595
77	GCF_000234725.1	*M. bovis*	Mexico
78	GCF_000934325.3	*M. bovis*	SP38
79	GCF_000967285.1	*M. bovis*	RD1
80	GCF_005155785.1	*M. bovis*	Danish delta-sapM
81	GCA_001544815.1	*M. microti*	12
82	GCF_904810355.1	*M. microti*	Maus IV
83	GCF_904810365.1	*M. microti*	94-2272
84	GCF_904810345.1	*M. microti*	Maus III
85	GCF_904810325.1	*M. microti*	OV254
86	GCF_904810335.1	*M. microti*	ATCC 35782
87	GCF_002982215.1	*M. microti*	ATCC 19422
88	GCF_000195955.2	*M. tuberculosis*	H37Rv
89	GCF_002116835.1 t	*M. tuberculosis*	Beijing-like/50148
90	GCF_000277735.2	*M. tuberculosis*	H37Rv
91	GCF_000667805.1	*M. tuberculosis*	H37Rv
92	GCF_014899985.1	*M. tuberculosis*	3-0096P6C4
93	GCF_009730215.1	*M. tuberculosis*	FDAARGOS_751
94	GCF_002116775.1	*M. tuberculosis*	Beijing-like/36918
95	GCF_009730235.1	*M. tuberculosis*	FDAARGOS_750
96	GCF_007833935.1	*M. tuberculosis*	MT-0080

No.	GenBank acc. no.	Species name	Strain ID*
97	GCF_001870145.1	*M. tuberculosis*	TB282
98	GCF_000016925.	*M. tuberculosis*	F11
99	GCF_000069185.1	*M. abscessus*	19977
100	GCF_003609715.1	*M. abscessus*	BD
101	GCF_001606295.1	*M. abscessus*	FLAC013
102	GCF_000497265.2	*M. abscessus*	JCM 15300
103	GCF_001677215.1	*M. abscessus*	FLAC055
104	GCF_001606215.1	*M. abscessus*	FLAC004
105	GCF_014843195.1	*M. abscessus*	JHN_AB_0032_1
106	GCF_001606255.1	*M. abscessus*	FLAC007
107	GCF_010731915.1	*M. abscessus*	JCM 30620
108	GCF_014843135.1	*M. abscessus*	JHN_AB_0004_2
109	GCF_014843175.1	*M. abscessus*	JHN_AB_0023_1
110	GCF_000770235.1	*M. avium*	DJO-44271
111	GCF_009741445.1	*M. avium*	DSM 44156
112	GCF_001865635.4	*M. avium*	OCU464
113	GCF_001936215.1	*M. avium*	H87
114	GCF_000014985.1	*M. avium*	104
115	GCF_004345205.2	*M. avium*	mc2 2500
116	GCF_005518035.1	*M. avium*	101034
117	GCF_003408535.1	*M. avium*	MAC109
118	GCF_002716925.2	*M. avium*	OCU901s_S2_2s
119	GCF_000219085.2	*M. avium*	S397
120	GCF_013357385.1	*M. avium*	DSM 44135
121	GCF_001941665.1	*M. caprae*	Allgaeu
122	GCF_002982225.1	*M. caprae*	ATCC BAA-824
123	GCF_014489215.1	*M. caprae*	NLA000201913
124	GCF_001369315.1	*M. caprae*	MB2
125	GCF_002105755.1	*M. colombiense*	CECT 3035
126	GCF_010730195.1	*M. conspicuum*	JCM 14738
127	GCF_002101675.1	*M. gordonae*	DSM 44160
128	GCF_013466425.1	*M. gordonae*	24T
129	GCF_001673255.1	*M. gordonae*	1245695.6
130	GCF_001417955.2	*M. gordonae*	CTRI 14-8773
131	GCF_001673475.1	*M. gordonae*	1275229.4
132	GCF_001675225.1	*M. gordonae*	GCF_001675225.1
133	GCF_001722405.1	*M. gordonae*	HMC_M15
134	GCF_000277125.1	*M. intracellulare*	ATCC 13950
135	GCF_002219285.1	*M. intracellulare*	DSM 44623
136	GCF_000418535.1	*M. intracellulare*	05-1390
137	GCF_002219265.1	*M. intracellulare*	ZUERICH-1
138	GCF_900116695.1	*M. intracellulare*	MC045
139	GCF_016755935.1	*M. intracellulare*	M.i.27
140	GCF_016756215.1	*M. intracellulare*	M026
141	GCF_016756135.1	*M. intracellulare*	M006
142	GCF_002285695.1	*M. intracellulare*	FLAC0133
143	GCF_002287605.1	*M. intracellulare*	FLAC0070
144	GCF_000723425.2	*M. marinum*	E11
145	GCF_003391395.1	*M. marinum*	CCUG20998

No.	GenBank acc. no.	Species name	Strain ID*
146	GCF_003609695.1	*M. marinum*	ATCC 927
147	GCF_003431645.1	*M. marinum*	DSM 44344
148	GCF_003431655.1	*M. marinum*	NCTC2275
149	GCF_000018345.1	*M. marinum*	M
150	GCF_003391415.1	*M. marinum*	1218R
151	GCF_016745295.1	*M. marinum*	MMA1
152	GCF_000164135.1	*M. parascrofulaceum*	ATCC BAA-614
153	GCF_002101845.1	*M. riyadhense*	DSM 45176
154	GCF_002116635.1	*M. szulgai*	DSM 44166
155	GCF_002102015.1	*M. xenopi*	DSM 43995
156	GCF_900453395.1	*M. xenopi*	NCTC10042
157	GCF_009936235.1	*M. xenopi*	JCM 15661T
158	GCF_000257745.1	*M. xenopi*	RIVM700367

* Type (reference) strains are marked with "[T]".

Suppl. Tab. 3. List of primers designed for the study.

Forward primer name	Sequence (5'-3')	Reverse primer name	Sequence (5'-3')	Set no.
F_3487509<br>F_25515551	CTCGCAATTGAGCCATGAG<br>TGTTGGGAGGTGGAAATCC	R_3487509<br>R_25515551	AGGCATGTGACGCAATTGC<br>CTAGGACGGTGCTGATCTA	1
F_3485935<br>F_21172891	ATGGACCAGCTGACGTTTG<br>CTGGGAAACCCGAGCATGA	R_3485935<br>R_21172891	AGGCATGTGACGCAATTGC<br>GCAATGTGTGGTGTCGTGCT	2[#]
F_3486704<br>F_25515551	CAGCTGACGTTTGACGTCT<br>TGTTGGGAGGTGGAAATCC	R_3486704<br>R_25515551	AGGCATGTGACGCAATTGC<br>CTAGGACGGTGCTGATCTA	3

[#] Selected primer set.

SUPPLEMENTAL FIGURES

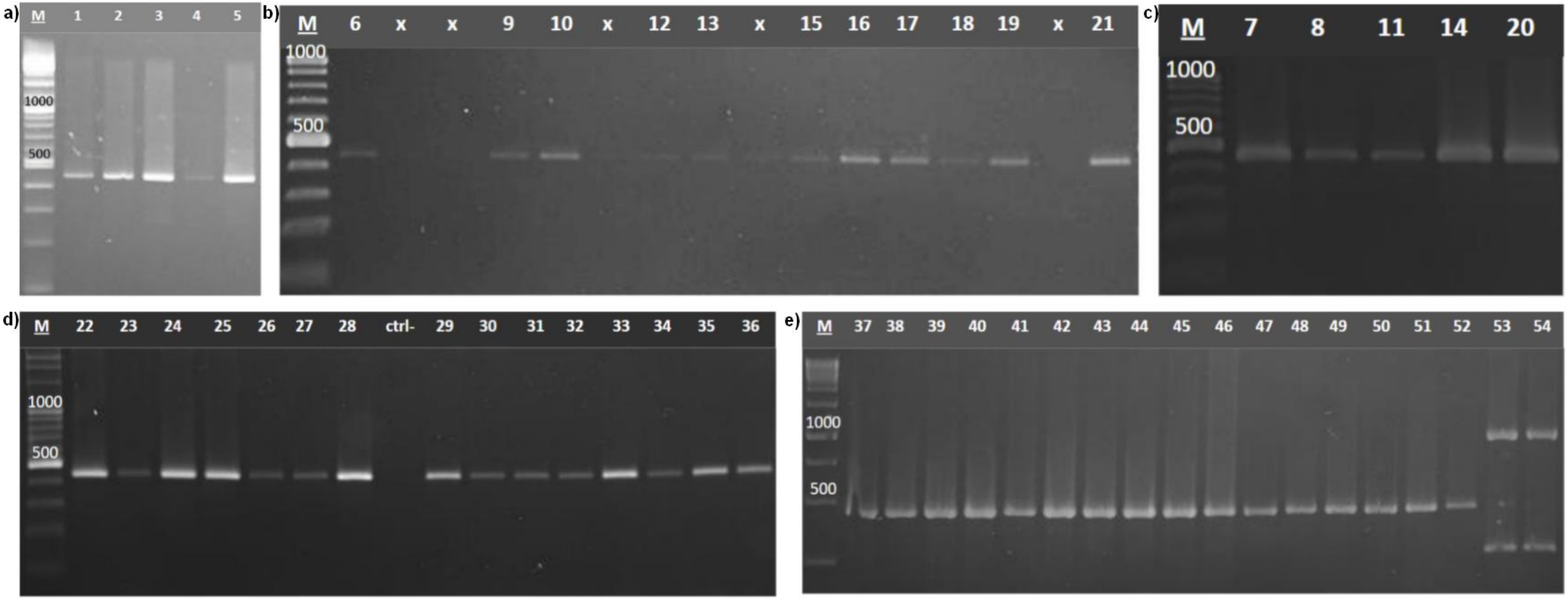

Suppl. Fig. 1. Electrophoretic visualization of the evaluation of a newly designed one-step multiplex PCR assay on *M. kansasii* strains. Lane numbers represent strain numbers of the MKC subgroup (see: **Suppl. Tab. 1**).

a-e) *M. kansasii* (expected band size: 450 bp)*;* **b) X** – due to the image compression, some bands were either not visible or barely visible in the captured photo. To enhance the signal, a separate gel (c) was run with a double load of the selected samples; **d) ctrl-** – negative control (no DNA); **a-d) M** – 100 bp Plus DNA Ladder; **e) M** – Low Range DNA Ladder; lanes 53-54 – atypical *M. kansasii* IIB.

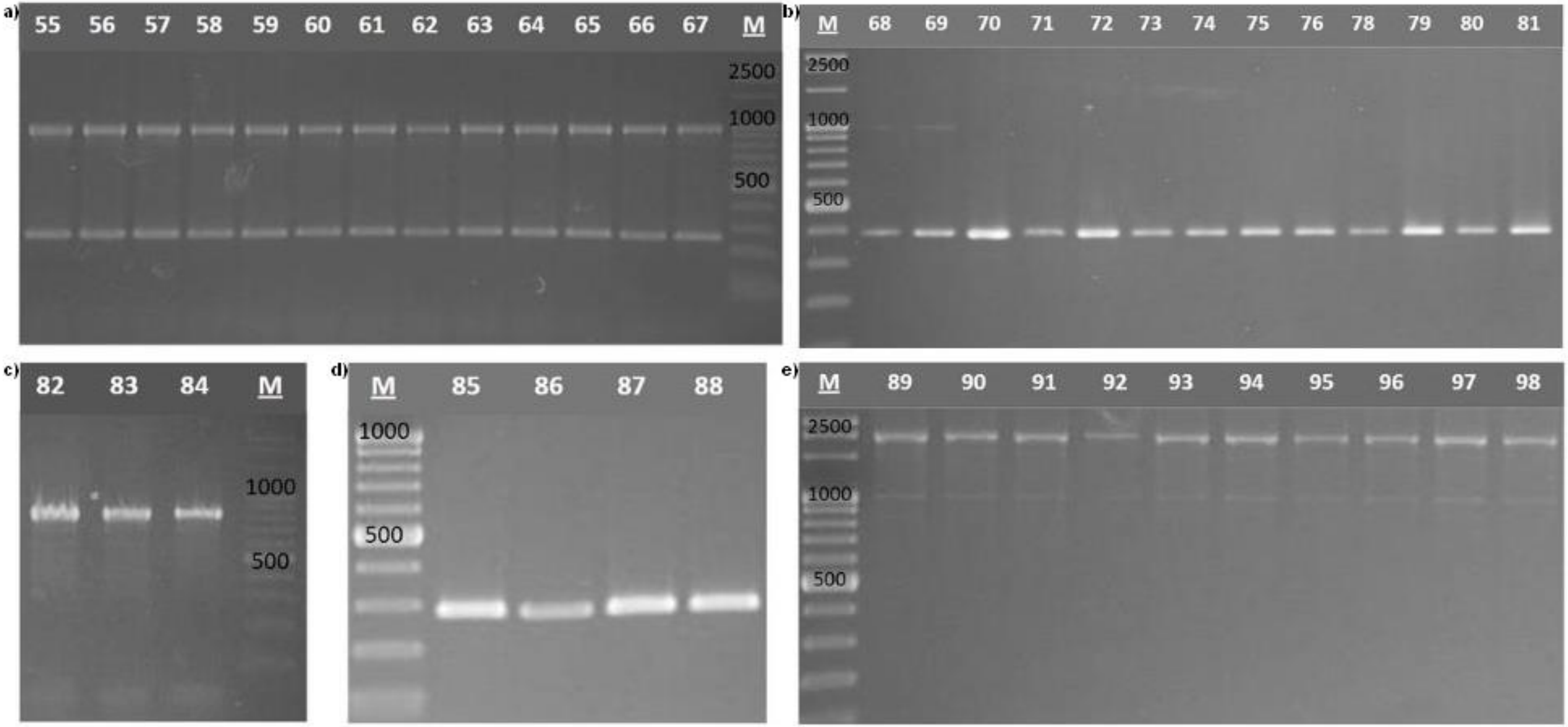

Suppl. Fig. 2. Electrophoretic visualization of the evaluation of a newly designed one-step multiplex PCR assay on strains of MKC species other-than *M. kansasii*. Lane numbers represent strain numbers of the MKC subgroup (see: **Suppl. Tab. 1**).

a) *M. persicum* (expected band sizes: 260 and 986 bp); **b)** *M. pseudokansasii* (expected band size(s): 392 (/and 985) bp); **c)** *M. ostraviense* (expected band size: 830 bp); **d)** *M. innocens* (expected band size: 287 bp); **e)** *M. attenuatum* (expected band sizes: 999 and 2200 bp); **a-e)** **M** – 100 bp Plus DNA Ladder.

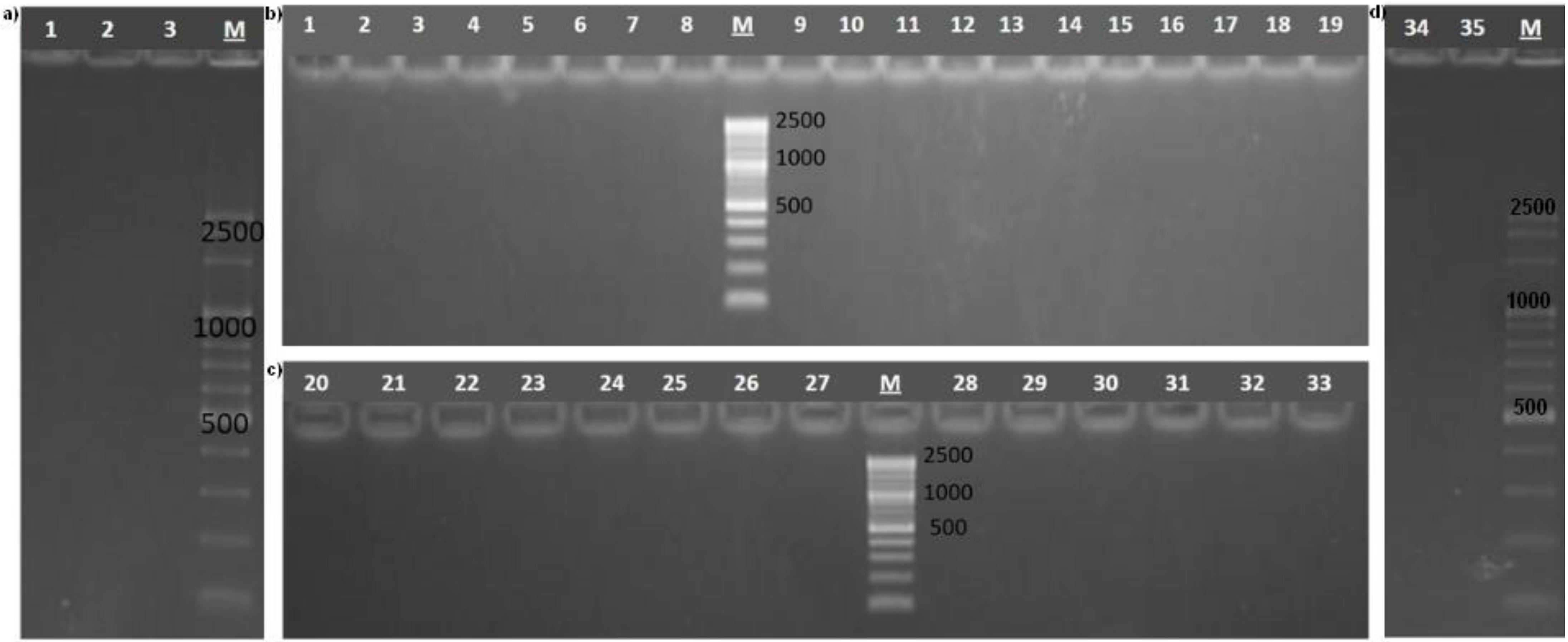

Suppl. Fig. 3. Electrophoretic visualization of the evaluation of a newly designed one-step multiplex PCR assay on non-tuberculous mycobacteria other-than *M. kansasii* complex (NTM other-than MKC) and *M. tuberculosis* complex (MTBC) strains. Lane numbers represent strain numbers of the MTBC and NTM other-than MKC subgroups (see: **Suppl. Tab. 1**).

a) *M. tuberculosis* complex; **b-d)** NTM other-than MKC; **a-d)** no bands expected; **M** – 100 bp Plus DNA Ladder.
